# “I’m a Mother Who Always Tries to Give My Children Hope”—Refugee Women’s Experiences of Their Children’s Mental Health

**DOI:** 10.3389/fpsyt.2019.00789

**Published:** 2019-11-01

**Authors:** Anna Pérez-Aronsson, Georgina Warner, Anna Sarkadi, Fatumo Osman

**Affiliations:** Department of Public Health and Caring Sciences, Uppsala University, Uppsala, Sweden

**Keywords:** child, mental health, mother, refugee, well-being

## Abstract

**Background:** The prevalence of mental health problems is high among refugee children. Childhood mental health problems have long-term negative consequences and costs both for the individual child and society. The present study aimed to explore refugee parents’ experiences of their children’s mental health.

**Methodology:** A qualitative explorative study was conducted. Data were collected through semistructured interviews with nine refugee mothers who have been in Sweden less than 5 years and with at least one child in the ages 8–14 years. Data were analyzed inductively using thematic network analysis.

**Results:** The global theme that emerged from the analysis was *Navigating the moving landscape of forced migration*, which described the refugee mothers’ experiences of the previous adversity the family went through, the ongoing transition in the new context, and, lastly, the pathways to promote their children’s mental health. Two organizing themes described mothers’ and children’s navigation of the forced migration: *Previous adverse events and new suffering* and *Promoting children’s well-being*. Mothers described aggression and frequent conflicts, or refusal to play or eat, in their children related to living conditions at asylum centres and social isolation. This improved when children started school and possibilities of social relations increased. Mothers’ own mental health and lack of language skills could also have a negative impact on the children. To focus on the present and have hope of the future was helpful to the children. Encouragement and social support from parents, teachers, and friends promoted children’s well-being.

**Conclusion:** The role of the host country in the promotion of the mental health of refugee children is emphasized. Interventions aimed to improve peer relations and reduce discrimination are needed, and these point to the school as a potential arena for positive change. Parental support groups were also mentioned as helpful in understanding the children’s need for support.

## Introduction

Sweden is a high-income country in northern Europe, with over 10,250,000 inhabitants (1). During the years 2000–2011, an average of approximately 26,300 persons per year sought asylum in Sweden (2). These numbers increased gradually to 81,301 asylum-seeking persons in 2014. In the following year, these numbers more than doubled; 162,877 persons sought asylum in Sweden in 2015. Among them were 70,384 children of whom 35,015 came with their families. The biggest group among these persons were Syrian citizens, constituting approximately one-third of all asylum-seeking persons in Sweden during 2015, followed by persons from Afghanistan and Iraq. In the aftermath of the drastic increase of refugees in 2015, the Swedish government took measures to reduce the number of asylum-seeking persons (3). The Swedish asylum law was limited to the minimum demanded by EU right and international conventions, and increased border controls were introduced. The new asylum law meant fewer possibilities to be granted asylum, temporarily permits of stay as a rule rather than permanent ones and limited possibility of family reunion (4).

Many women who have faced forced migration are mothers. Not only is it likely women in these families have encountered profound and multiple traumas (5), it is likely their children have too (6). Traumatic events before and during migration are associated with an increased risk of mental health problems such as post-traumatic stress in refugee children, as well as emotional and behavioral problems. Yet, current life circumstances in the receiving countries, such as asylum status, school attendance, and perceived discrimination, can be of equal or greater importance to refugee children’s mental health than previous experiences of violence (6). Recent studies have also found a connection between refugee caregiver’s psychopathology and poorer mental health in their children (7, 8). Refugee mothers, thus, have a double burden: dealing with their own mental health problems, knowing all too well that their children may be affected, and trying to support their children in adaptation and dealing with mental health problems (9). According to a systematic review on the prevalence of psychiatric disorders and mental health problems among refugee and asylum-seeking minors in Europe, up to a third could be affected by either depression, anxiety disorders or other emotional or behavioral problems, and up to half by post-traumatic stress disorder (PTSD) (10). Childhood mental health problems are associated with lower academic achievement (11), increased risk of psychological problems as an adult and a substantially reduced income (12). Drawing on this, developing successful early interventions for refugee children might have the potential to reduce suffering and cost for the individual child as well as the society at large. Yet, the Swedish mental health care resources are not sufficient for refugee and asylum-seeking children’s need (13). There is a need for interventions that prevent mental health problems and are successful in reaching all refugee groups (13).

When parenting a young person with mental health problems, there is a moral imperative to care for the child but this is challenged by difficulties in coping with the extended parental responsibility. Parents’ knowledge of mental health conditions, their interactions with healthcare professionals, and their relationships with their child are also important (14). It is likely that the refugee context exacerbates these issues, as the cultural narrative around mental health conditions and healthcare systems are different (15). Refugee families can be difficult to recruit and retain in interventions (16). However, culturally tailored interventions adapted to the families’ needs can be more effective (e.g., 17, 18). Moreover, refugee mothers in a wide variety of settings have demonstrated impressive resilience and an ability to negotiate cultural, language, and geographical barriers to support their children’s adaptation (19). Thus, understanding refugee mother’s experiences of their children’s mental health is instrumental in developing indicated prevention strategies for refugee children at risk of prolonged mental ill health, a necessary component in achieving the United Nations’ Sustainable Development Goal of Good Health and Well-Being.

According to a recent review (20) on the health needs of refugee children in Europe, the majority of existing literature on the mental health needs of refugee children is from North America and Australia. Furthermore, there is to the best of our knowledge, no Swedish study conducted after the restrictions in the Swedish asylum law (4) that explores how refugee mothers in Sweden perceive their children’s mental health in resettlement in Sweden. Literature from other parts of the world suggest that certain health risks and needs are shared by refugee children in the world; however, the specific health needs and risk depend on conditions both before and during migration as well as in resettlement, such as health care policies in the receiving country (20). There is a need of more knowledge on the mental health needs of refugee children in Europe, particularly concerning effective promotion and preventive services, early recognition, access to care, and intervention. There has been exploratory research on group-based therapy programs with unaccompanied refugee youth in Sweden (21), but this works need to be extended to younger children arriving with their families. This study aims to contribute to increased understanding of how refugee mothers, coming to Sweden since the restrictions in the Swedish asylum law was introduced, experience their children’s mental health needs. Such knowledge may be valuable in order to adequately shape promoting and preventive interventions for refugee children’s mental health.

### Aim

The aim of the study was to explore refugee mothers’ Sweden experiences of their children’s mental health.

## Methods

The study design was an explorative qualitative study with inductive approach. A qualitative study design is appropriate when the aim is to understand people’s experiences of and perspectives on a phenomenon (22). As only a few Swedish studies were found on the research area of interest, an explorative study was considered appropriate as it allows the researcher to follow up on the participants’ answers and seek new information around relevant issues (23). Ethical approval for the study was obtained by the regional Ethics Committee (Dnr 2018/486).

### Participants

Nine refugee mothers participated in the study; six from Syria, two from Afghanistan, and one from Iraq (see **Table 1** for further demographic characteristics). The inclusion criteria were (i) to have lived in Sweden for less than 5 years and (ii) have at least one child between the ages of 8 and 14 years at the time of recruitment. The criteria were chosen to align with a planned randomized controlled trial evaluating a trauma-focused group intervention for refugee children. The current study was conducted prior to the randomized controlled trial to understand child mental health needs and perception among the target population (refugee families with children 8–14 years old) and to inform intervention delivery and trial procedures. Recruitment was conducted by the first author using purposive sampling. Purposive sampling is appropriate to find information-rich cases which can lead to insights and in-depth understanding of relevant issues (22). The mothers were recruited through community sites known to have a high proportion of refugee and newly arrived families. Three were recruited through language schools, three through nongovernmental organizations, and one through an open preschool. Two additional participants were recruited through snowball technique. Snowball sampling can be useful when it proves difficult to reach the target population (22). The participants’ median age was 35 years, their median number of children was 4, and the median time in Sweden was 3 years (see **Table 1**).

**Table 1:** Demographic characteristics of the participants.

Variable	Participants = 9
*Age*	22–48 years (Md 35 years)
*Gender*	9 females
*Time in Sweden*	1.5–5 years (Md 3)
*Number of children*	2–5 (Md 4)
*Age of children*	1–17 years (Md 9.5 years)
*Marital status*	7 married, 1 widowed, 1 separated
*Occupation*	7 studying, 2 parental leave
*Country of origin*	6 Syria, 1 Iraq, 2 Afghanistan
*Permit of stay*	7 yes, 2 rejected

### Data Collection

Data was collected through individual interviews, using a semistructured and study-specific interview guide (**Table 2**). The guide covered three topics relevant to the aim of the study: (i) parents’ experiences of their children’s mental health since arriving to Sweden; (ii) any challenges for the children’s mental health; and (iii) any support the children had received or the parents believed could be helpful for their children. Before data collection commenced, the research plan and the interview guide were presented to researchers specializing in refugee children’s mental health, as well as to representatives from the study population for feedback. A pilot interview was conducted to further assess the interview guide. No major change was applied to the interview guide after the pilot interview, which was included in the analysis. All interviews were conducted in a calm, secluded environment that had been chosen together with the participants. In eight of the interviews, a professional, authorized interpreter from an agency was used (one Kurmanji-speaking, two Dari-speaking, and five Arabic-speaking). In six of those interviews, a contact interpreter was used, but in two, a telephone interpreter had to be used as the interviews were conducted in a remote area. All interpreters were females. In three of the interviews, it was possible to use the same interpreter. One participant did not wish to use an interpreter but choose to conduct her interviews in English. The duration of interviews was between 25 and 95 min, with an average of 65 min. All interviews were recorded on a digital audio file with consent from the participants, assigned a code, and transcribed verbatim.

**Table 2:** Interview guide.

Topic	Questions
Mothers’ experiences of their children’s mental health since arriving in Sweden	-How has your child felt since you arrived in Sweden?
	-How do you perceive mental health?
Any challenges for the children’s mental health	-Please tell me about any difficult situations that your child has handled well?
	-Tell me about a situation that has been challenging for your child?
	-Are there any other challenges that you believe children coming to Sweden could need help or support with?
Any support that the children had received or that the mothers believed could be helpful for their children	-Was there anyone or anything that helped your child, or you as a mother, to handle this difficult situation?
	-What kind of support do you think that children coming to Sweden need?

### Data Analysis

The transcribed interviews were analyzed inductively using Attride-Stirling (24) thematic network analysis method. Thematic network analysis was chosen as this method “allows a sensitive, insightful, and rich exploration of a text’s overt structures and underlying patterns” (24), p. 386), thus addressing both subjective experiences and the underlying context. The transcripts were read several times to get a sense of the whole data. The text that captured parents’ experiences of their children’s mental health were highlighted and extracted into a table in a word file, which were then coded. The codes focused the core meaning of the text segments and related to the aim of the study. After this step, codes were compared to reveal similarities, differences, and recurring patterns. Codes with similar meanings were brought together into basic themes. The basic themes captured the phenomenon that participants described, i.e., parents’ experiences of their children’s mental health and the support parents believed could be helpful for their children. All basic themes with similar meanings were grouped into two organizing themes. In the last step of the analysis, an overarching global theme that captured the main metaphors in the text was identified (see **Table 3** for an example of the process of coding data).

**Table 3:** Example of the process of coding data and placing data in themes.

Text passage	Code	Basic theme	Organizing theme
Here in Sweden, we have also moved several times, so she does not want to make friends and acquaintances because she had to let go of her friends in our country and she came here, and we have moved a few times like I said, so she does not want to always be making new friends and letting them go. (IB)	Separations due to frequent relocations	The impact of societal factors	Previous adverse events and new suffering
They are bullied in school. For example, my oldest daughter suffers because the other children pick on her all the time because her Swedish is not that good. Because they lived here before her and they know some more Swedish language than her. (IE)	Peer problems in school	Difficulties in social relations	Previous adverse events and new suffering
He got help from his friends that speaks Arabic, they helped him with the language and with playing with others/…/He feels safe when one of his friends speaks both Arabic and Swedish. (ID)	Friends that speaks the same language are helpful	The importance of supportive relationships	Promoting children’s well-being

The analysis was a back-and-forth process until consensus was achieved. To verify the accuracy of the themes and the placement of data in themes, comparisons were made throughout the analysis between all text data and the themes and codes (25). Relevant quotations were selected to illustrate and support the content of the themes.

## Results

**Figure 1:**
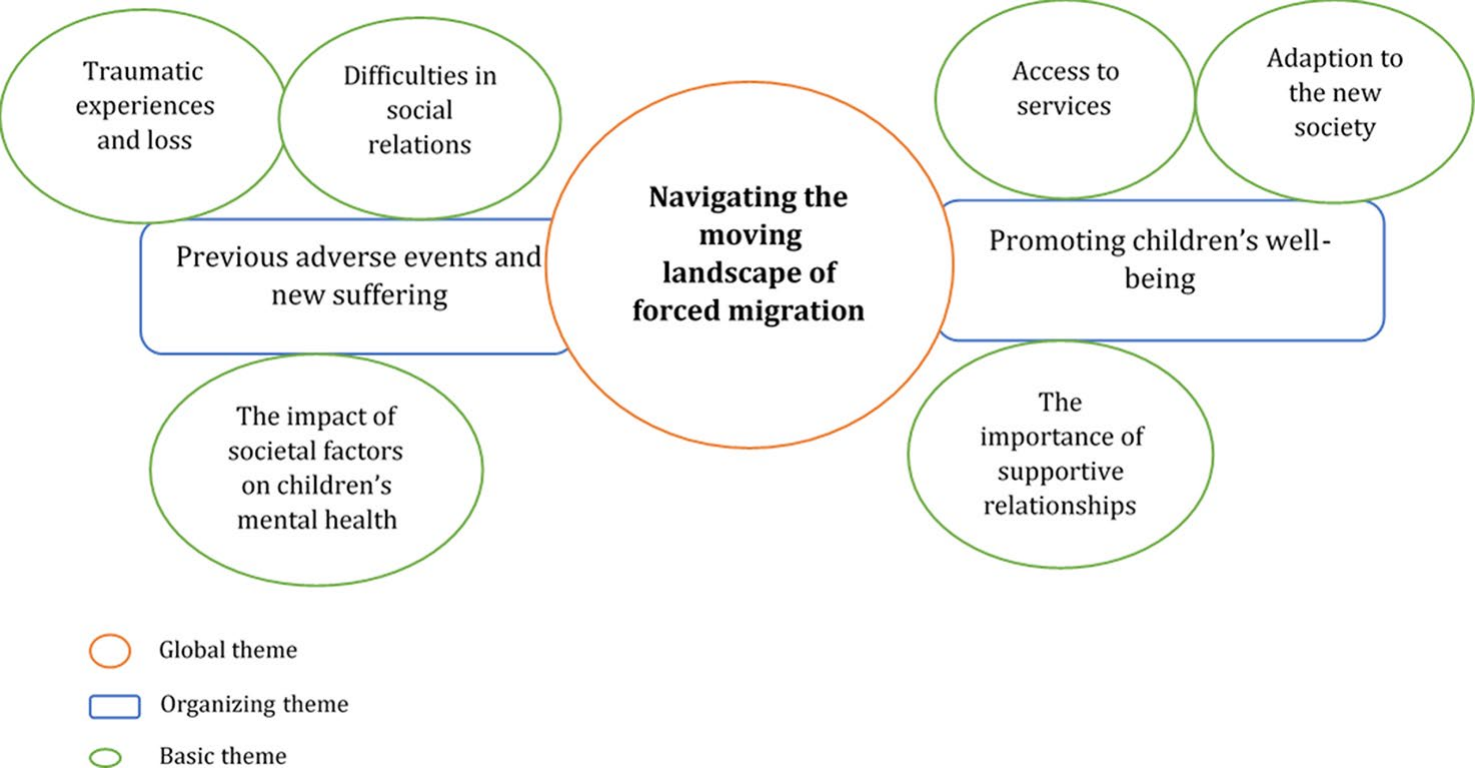
Refugee mothers’ experiences on their children’s mental health.

The global theme that emerged from the analysis, *Navigating the moving landscape of forced migration*, was a metaphor that captured refugee mothers’ experiences of the previous adversity the family went through, the ongoing transition in the new context and lastly the pathways to promote their children’s mental health. Two organizing themes described mothers’ and children’s navigation of the forced migration: *Previous adverse events and new suffering* and *Promoting children’s well-being* (**Figure 1**).

### Previous Adverse Events and New Suffering

The organizing theme of previous adverse events and new suffering captured refugee mothers’ experiences of their children’s difficulties since arriving in Sweden, with regard to both emotional and behavioral problems and what they believed to be causing or contributing to these problems. Three basic themes explained parents’ experiences: *traumatic experiences and loss; difficulties in social relations*; and *the impact of societal factors on children’s mental health*.

#### Traumatic Experiences and Loss

Memories of traumatic experiences of violence, war, and dangerous circumstances during the flight were brought up as something that caused the children suffering upon arrival. Refugee mothers described how their own traumatic experiences and loss of friends and the extended family affected children’s mental health and also how mothers were affected by their children’s mental health problems.

Refugee mothers had noticed several difficulties in their children related to the previous trauma. Children had shown fear of separation from parents, due to having been separated from them during the migration or due to having witnessed violence and being scared of strangers. Fear of strangers was also described in children as a consequence of witnessing violence and abuse, but also as something that children had learned in their home country, to be cautious and avoid strangers due to the insecure situation. Some mothers had experienced sleeping difficulties or bed-wetting in their children, flashbacks, fear of the dark, or nightmares. One mother expressed that the children’s ages seemed to affect how much these memories troubled them after arrival in Sweden.

*The youngest son had a very tough time when we came to Sweden. It was because he saw so much on the way here. He saw the smuggler beat his dad, my husband, and this affected him. So when we came to Sweden he met with a therapist, a counsellor he could talk to, and with time we experienced improvement, that he was doing better. The two older were a little smarter, they understood that “I have come here, and it is safe here”. So they did not feel as poorly. But the youngest, he felt very bad, he was very scared./…/So in the beginning when someone wanted to hug him, or talk to him, he ran to me*. (Mother of four children: 1, 7, 8, and 10 years old)

Refugee mothers described how their own traumatic experiences of the war and flight had affected their own mental health, which, in turn, had affected the children.

*I lost my father and sister in the sea in Greece/…/I was doing very poorly at the asylum centres, but I have gotten much better/…/And it’s kind of obvious, if a mum or dad is not feeling well, it will automatically affect the children*. (Mother of four children: 1, 7, 8, and 10 years old)

In contrast, children’s mental health problems also affected mothers’ mental health, as another mother explained:

*Before she used to pee in her bed… (sighs). Because she was tired when she slept and she could not wake up. I put the clock, and I fix it. When she slept, I would wake her up after one hour and I would take her to the bathroom. And then again after one hour. And if I forgot her … she would pee in the bed. Because she was afraid*. (Mother of two children: 11 and 13 years old)

Moreover, several refugee mothers described that their children had experienced grief and feelings of emptiness and void upon arrival in Sweden, due to having left behind their friends and relatives, schools and homes, and toys.

A process over time was described with regard to children’s difficulties due to previous traumas. Refugee mothers had observed an improvement in their children at the time of the interviews, in some cases, after having been referred to a counsellor. Nevertheless, it was mentioned that children’s memories could still resurface and trouble them.

#### Difficulties in Social Relations

Difficulties related to social relations were mentioned by several parents as an important part of their children’s problems. Refugee mothers described changes in how their children acted towards others after arriving in Sweden, compared to in their home countries before migration. Some had experienced increased aggression and more frequent conflicts with others in their children and increased defiance against parents. The increased defiance was described as particularly challenging for mothers to handle. Some mothers described that their children wanted contact with others, but they were shy and scared to contact others. Not having contact with others was described to make the children sad and create feelings of loneliness and of not being liked by others. On the other hand, parents described that some children seemed not to want to engage with others and showed withdrawal and isolated themselves from social interactions.

*I tried many times. When there was a friend at my house, I tried to make the oldest girl sit with us and talk or even dance. She always refused. She did not want to. She did not want to laugh, and she did not want to dance. She wanted to isolate herself and be alone in her room*. (Mother of two children: 13 and 14 years old)

Language-related and cultural barriers were brought up as partly contributing to isolation. Not knowing the language was also described as something that caused the children feelings of insecurity and worry.

*At the beginning, it was a bit difficult for my child to go to school and talk to the teachers there. Everyone spoke Swedish; no one spoke Arabic. He asked all the time: how will I tell them if I need something? If there had been an Arabic-speaking teacher or an Arabic-speaking assistant that could help those who are newly arrived. For example, my child would come home many times in pain and he could not tell the teacher he was in pain*. (Mother of two children: 1.5 and 9 years old)

Although language difficulties were brought up as an important contributing cause to isolation and loneliness, all problems with social relations had not improved with time and increased language proficiency. Perceived discrimination from Swedish children—and in some cases, teachers—was described by some mothers, making the children feel unliked and unwelcomed. Refugee mothers described that bullying and peer problems made their children sad and affected their self-esteem and led to them not wanting to go to school. Although several mothers shared experiences of teachers as a resource that had been important for their children, in this context, it was described that parents and their children had experienced a lack of support from teachers and schools.

Furthermore, lack of social networks in the new home country and struggling to develop new relationships were described by mothers to have caused their children substantial suffering. Children were described to have reacted to this isolation by becoming increasingly passive and difficult to activate.

*I brought them to the park, to the playground. They just sat down there and held their hands in front of their … I told them to play and they said, “but we don’t have any friends to play with.” They did not want to go outside at all, I forced them to go outside with me. There was a park near us; I would force them to go down to the park with me. They would say, “what are we doing there, there is no one who can play with us, no one who can talk to us”*. (Mother of four children: 8, 12, 14, and 15 years old)

#### The Impact of Societal Factors on Children’s Mental Health

Refugee mothers brought up several societal factors that had a negative impact on their children’s mental health. Living in asylum centres and long waiting time before starting school, frequent relocations, parents not getting access to language classes, and lack of social network were described as particularly harmful for the children’s mental health.

Mothers described that the living conditions at asylum centres with cramped housing accommodations, restricted freedom to move, and lack of activity and language barriers with other children at the camp had worsened children’s difficulties with aggression and frequent conflicts and delayed time to recovery from traumatic experiences. Some had experienced that their children had reacted to the difficult living conditions in the asylum centres by becoming more passive or even refused to eat. Mothers also mentioned that living in the asylum centres made it more difficult for the parent to be a good support for their children.

Long waiting times before starting school, in some cases more than 6 months, were experienced as an aggravating circumstance for the children’s mental health, as it meant the children were not given the opportunity to learn the language or to have a place to meet friends or a meaningful activity.

*They were home for a while, almost 6–7 months. There was no school and no possibility to go outside. No friends, nothing. They were almost completely isolated. My youngest daughter was strongly affected by this. She was five years, then. It got to the point that she stopped eating*. (Mother of four children: 8, 12, 14, and 15 years old)

Some mothers mentioned that they were not entitled to SFI (language courses) until having received a permit to stay, which in some cases had taken more than a year. Some mothers had still not received a permit of stay and thus were still not entitled to SFI. Both mothers with and without a permission to stay described their own lack of language proficiency as a potential risk to their children’s health, as it made contact with services such as schools or health care difficult. Furthermore, mothers without permission to stay in Sweden shared experiences of the families’ insecure asylum status having a negative impact on the children’s well-being, particularly for the oldest siblings. Bullying in school due to insecure asylum status was described, as were experiences of interrogations at the Migration Agency bringing back children’s memories of traumatic experiences, causing anger and difficulties concentrating in school. Relating to the impact of the asylum status on children’s well-being, mothers without permission of stay described that they themselves were currently struggling with mental health problems, needing treatment from psychologist, due to the burden and worries that their families’ insecure asylum status caused them. The mothers expressed that their mental health problems, in turn, affected their children, as it became more difficult for the mothers to provide the support and the presence that they felt they ought to as parents.

### Promoting Children’s Well-Being

The organizing theme, promoting children’s well-being, captured parents’ experiences of improvements in their children’s mental health and well-being since arriving in Sweden. The theme included perceived strengths in the children as well as factors refugee mothers experienced to promote their children’s well-being or improve their mental health problems. Four basic themes were identified: *access to services*, *adaption to the new society*, *the importance of supportive relationships*, and *focusing on the present and hoping for the future*.

#### Access to Services

Access to different services in the community was brought up as positive for the children’s mental health. Receiving a permit of stay and starting school or preschool was important, as it increased children’s access to services and gave life a sense of normality. Even mothers without a permit of stay had experienced an improvement in their children’s well-being, related to when they had left asylum centres for another accommodation and their children had started school which suggests that, for these accompanied refugee minors in Sweden, school access can improve well-being regardless of asylum status. School was experienced to promote the children’s well-being in many ways.

*It was difficult for the first year when they came here, but it started getting better after that/…/They do a lot of activities together now, not like before/…/These conflicts that they had before, with other kids, they’re gone too. They can play with others as well as before. They play all the time. They talk a lot about school, that they’re happy, that they’re going to invite their friends home and that kind of stuff. When they’re going on some excursion they’ll talk about that day all week*. (Mother of five children: 2, 5, 8, 10, and 12 years old)

School also meant meeting teachers, which mothers described as often supportive and attentive to the children’s needs. In some cases, teachers had even recommended parents to seek mental health care for their children. Even routine health checkups proved to be important.

*We met with the school nurse. It was a routine control of the vision, the back, weight, height, and those things. At the same time, she asked how the kids were doing. And she asked us what we had been through, about the journey here, how are kids were doing, did we experience any physical abuse, violence, assault, if we had seen anyone killed or beaten in front of us, everything. So I told her everything about how my kids were doing, and she said that she would book an appointment with a counsellor so we could talk to them. It is great if newly arrived parents get those kinds of offers*. (Mother of four children: 8, 12, 14, and 15 years old)

Some mothers expressed that they had not initially understood their children’s needs, or which help they could seek. Parenting support courses were mentioned as needs parents have, as it could help parents to understand their children’s needs in the new country and give them knowledge on how to better handle those needs.

#### Adaptation to the New Society

Refugee mothers described that their children had the ability to adapt quickly to their new surroundings, which in their experiences strengthened and promoted their children’s well-being. Children realized they were in a safer place than before migration, and problems such as fear and anxiety diminished.

*She is better than before. She was sad before, all the time. She was afraid. She could not go to another room alone in my country. Because she was afraid of the {war} noises/…/. When she came here, the first time she said “mum, I want to go to the toilet, come with me.” The first time I took her. The next time I took her, and left her alone. Now she goes and she sits there alone. And she goes outside./…/She sees that everything is safe, everything is ok./…/Now she can go alone, to the grocery store, to the shop*. (Mother of two children: 11 and 13 years old)

Refugee mothers experienced that, compared to their children, they often had greater initial difficulties to adapt to the new society and have contact with others; however, they dared to do that for the sake of their children. Some mothers expressed that it was important for children’s well-being that also the parents could find a way to adapt to the new society.

*I did not know anything. I treated him like I would in my home country; he is a child so he maybe just needs food and a roof over his head, just that/…/But then with time you start to adapt, change, and then transfer these little changes to the child as well. Think differently. So if you want to find or help the child you should start with the parents first*. (Mother of four children: 2, 4, 8, and 9 years old).

Relating to this, some mothers discussed the cultural differences between Sweden and their home country with regard to the view on mental health problems and health seeking behavior. According to the mothers, mental health problem or seeing a psychologist was embarrassing, even stigmatizing, in the home country (Syria) and could cause trouble such as a girl not being able to marry. However, the mothers said that, in Sweden, it was perceived “normal” to seek a psychologist. Some mothers described a process over time in their own view of mental health problems; they shared that, when they newly arrived, they would not have sought mental health care for their children. However, after having been advised to seek health care for their children and having had positive experiences of psychologists or counselors, they would now recommend parents, whose children were struggling with similar problems as their own children had, to seek health care. This process over time was not shared by all participants. Some mothers said that they themselves are responsible for helping their children and that they would perhaps ask other parents for advice but they would not seek health care.

#### The Importance of Supportive Relationships

There were several relationships that parents highlighted as crucial and supportive for the children’s well-being. Refugee mothers regarded themselves as an important source of support for their children. To spend time with a parent was mentioned as important for children experiencing difficulties. Encouragement from parents was stated to increase children’s self-esteem. One mother also mentioned that it was important for the children’s well-being that the parents had a positive tone in their own relationship.

*I try to cheer her up. Here is an easy example: if I have homework from my school, you know I can take a picture of it and translate it with Google translate. But I call out and ask for her help: “can you come to help me?” I could do that in five minutes on my phone. But I call for her, and she becomes happy [laughs], she gets really happy. She will tell her oldest sister, “Look I’m much better than you, I’m helping mum with her homework.”* (Mother of four children: 8, 12, 14, and 15 years old)

Refugee mothers expressed that having friends to play with who could speak both the children’s mother tongue as well as Swedish could make children feel more secure and help them build new friendships.

*To contact others with the language, that was the hardest. When he wanted to play with others, he could not tell them./…/He had some help from his friends who speak Arabic. They had to help him with the language and it got better./…/He feels safe when one of his friends can speak both Arabic and Swedish*. (Mother of two children: 1.5 and 9 years old)

A parent with some knowledge of Swedish was described as an important support for the rest of the family, as it could make contact with the surrounding society easier. Furthermore, having a relative in Sweden that had been here for a few years was mentioned as potentially important for the children’s mental health and access to support. Mothers said that they would first turn to a friend or another parent for advice if their children had some needs. A relative with knowledge about Sweden and the available resources had thus been an important support in some cases, as the relative could recommend parents to seek mental health care for their children or give them advice on parenting in the new country. Teachers who were refugees and understood the children’s difficulties were also described as important support. The mothers in the study who had taken their children to services such as psychologists or counselor described that it had been after being advised by a relative or the child’s teacher. One mother expressed that she had not known anything about the available mental health services and that she doubted that other newly arrived parents would seek mental health care services for their children if no one would ask them or advise them. This illustrates how the mothers’ relationships and interactions with other adults can influence the children’s well-being.

#### Focusing on the Present and Hoping for the Future

Refugee mothers had different views on how to handle their children’s memories of the past, their present, and their future. Some mothers described that they tried to distract their children from difficult memories and reminded them of positive memories, while other mothers felt children needed to forget about the positive memories that they had left behind as well to be able to focus on starting a new life in the new country. Mothers expressed that when children were troubled by previous difficulties it helped them to focus on the present and taking joy in their possibilities in Sweden.

Several mothers emphasized allowing children to dream about and have hope for the future as something that could help the children to cope with previous traumas and new suffering. Refugee mothers described that children should be allowed to dream about life in Sweden, but also about once again revisiting their previous home.

*I try to talk about the future all the time, not the past. That it’s something that is over, you can’t dwell on that. Start with today, one could say. To just think positive. To not think about the negative things that have happened*. (Mother of five children: 2.5, 10, 12, 15, and 17 years)

*I am a mother who always tries to give my children hope and support them. My oldest child says “I want to be a doctor here in Sweden.” The other says “I want to be a prosecutor.” So, I support them. They are so young*. (Mother of four children: 1, 7, 8, and 10 years)

It was also described that it was their children and husbands that gave refugee mothers’ hope for the future, which contributed to improvement of their own mental health.

*It was for the sake of my husband and my children that I got hope for the future, and felt better. Sometimes, you get tired of life but because of my husband and children I got hope, and then you can continue and fight for this life*. (Mother of four children: 1, 7, 8, and 10 years)

## Discussion

### Key Findings

Refugee mothers stated that their children were affected by the trauma of the war and dangerous circumstances during the flight. The refugee mothers’ own trauma impacted their children’s mental health, and the children’s mental health problems had an effect on mothers’ mental health as well. Living in asylum centres, the asylum process, frequent relocations, and lack of access to school worsened children’s mental health problems. The children’s trauma experiences made it difficult for them to make friends, and they frequently had conflict with other children and parents. Even though difficulties with trauma experiences improved over time, problems with social relationships did not always disappear and peer problems, such as perceived discrimination, remained an important issue for the children’s mental health. Refugee mothers emphasized that access to school improved children’s mental health and gave them something meaningful to do. Parental support groups were mentioned as helpful in understanding their children’s need for support. Focusing on the present and the future was described as contributing to children’s and parents’ well-being, as there were supportive relationships within the family and making new friends.

### Methodological Considerations

Trustworthiness of the study is discussed using the four criteria of Linclon and Guba (26) that are dependability, credibility, confirmability, and transferability. The dependability of the study was enhanced by thorough documentation of the process of study planning, recruitment, data collection, and analysis. The interviewer (APA) has relevant personal experiences. She is a child of a refugee parent as well as a parent of three small children. This might have created biases, such as how questions were asked, and there could be a risk of not asking certain questions or that the researcher might seek to confirm her own experiences. However, the use of an interview guide contributed to credibility. The interviewer’s own experiences could also have been an advantage, as they might have facilitated contact with the participants as well as have made it possible to ask insightful questions, thereby contributing to information-rich data. To ensure the credibility and confirmability of the study, the first author conducted the analyses and the last author reviewed the codes and basic organizing and global themes. All authors have also discussed together until consensus was reached.

One important potential limitation was the use of interpreters. When using interpreters, meaning can get lost in translation, which could affect credibility and confirmability. It is important that interpreters not only understand what the researcher is asking but also to give complete verbatim translations of the responses. Professional authorized interpreters were used in the present study to ensure a certain degree of competence in the translations. Trustworthiness was enhanced by the researcher ending each interview with a short summary of what had been described, to check if it had been correctly interpreted or if there was any misunderstanding.

This study included a small group of mostly Syrian refugee mothers living in the middle of Sweden, and so, the results cannot be transferred to all refugees in Sweden but could point to how a similar group of refugee parents experiences their children’s mental health needs. Participants of different ages and number of children were recruited, and efforts were made to include mothers from urban and rural areas, who had and had not yet been granted a permit of stay, to increase the breadth of experiences. Despite the relatively small number of participants, the interviews generated rich data. Analysis was conducted in parallel to recruitment and demonstrated that participants’ experiences were similar.

### Implications of the Findings for Practice, Policy, and Research

To the best of our knowledge, this is the first Swedish study exploring how, mostly Syrian, refugee mothers perceive their children’s mental health during the first few years since arriving to Sweden. Previous research shows that refugee children’s mental health is affected both by past traumas and by difficulties in the new country, related to challenges such as discrimination, refugee policies, and lack of resources (6). This study provides increased understanding of how refugee mothers in Sweden experience such pre- and post-migration factors and how they understand them to affect their children’s mental health.

The findings align with international research on refugee experiences of parenthood in that resettlement difficulties are described as contributing to adversity in addition to events prior to migration; drawing on the resources of the host country and focusing on hope for the future is described as important; and children are portrayed as giving meaning to life (19). Although supportive relationships within the family were described as important in the present study, it seems that, when discussing children’s mental health, there is less emphasis on transnational ties, whereby links are maintained with the home country, than reported in previous studies with refugee parents (19). This could relate to cultural differences in recognition and understanding of mental health and emphasizes the role of the host country in the promotion of the mental health of refugee children.

The present study highlights the significance of considering the mothers’ needs and resources when developing and implementing interventions aimed to prevent refugee children’s mental health problems. This supports the provision of parenting programs designed to assist displaced parents in understanding their children’s mental health and providing guidance on how they can provide support (e.g., 17, 18). Previous research indicates that such parental support for refugee families is associated with improved child behavior (17).

The findings call for policy makers to address societal risk factors that can adversely affect refugee children’s mental health. Practical examples of this include working to decrease the number of relocations, as well as reducing the waiting time for school placements. The interactions between different factors, such as how the asylum process can affect school performance or how living conditions can delay recovery from trauma, suggest that a multifactorial approach to mental health interventions could be valuable.

A key finding from the present study was that social difficulties continued to cause the refugee children suffering in resettlement, even after problems due to previous trauma had improved. This emphasizes the need for interventions aimed to improve peer relations and reduce discrimination and points to the school as a potential arena for positive change. This supports previous research with children residing in an asylum center in Sweden, which revealed the importance of school. The refugee children conveyed strong feelings of love and happiness for school, but reported issues relating to misrecognition by Swedish peers and how this can be aggravated by systematic segregation, such as placement into “introductory classes” (27). Another study, exploring Somali adolescents’ experiences of integrating into Swedish society, uncovered perceived discrimination by teachers and reports on the discord between refugee parents’ knowledge and perceptions and the Swedish school system (28).

The school can also act as a gateway to other important services. For example, the present study uncovered the potential importance of routine health checks. The integration of trauma-related questions into health checks, by school nurses or across healthcare services more broadly, could help to identify those children who would not otherwise have sought mental health care. Cultural differences in health seeking behavior were described by some mothers, and thus, accessing services *via* school may be considered more acceptable than visiting a psychologist, which is potentially stigmatizing.

The wealth of practical suggestions arising from the interviews with refugee mothers demonstrates the potential value of user involvement in the design and implementation of practice and policy. Future research could examine the impact of user involvement in designing and evaluating mental health interventions for refugee children. Further studies aimed at exploring other family members’ perspectives, such as fathers or extended family members, could give additional value, as well as exploration of refugee children’s own perspectives on their mental health.

### Conclusions

The present study explored refugee mothers’ perceptions of their children’s mental health. It highlights the significance of considering the mothers’ needs and resources when developing and implementing interventions aimed to prevent refugee children’s mental health problems. When placing the findings in the context of existing literature, previously reported transnational coping strategies seem less prominent, which may be due to cultural differences in the recognition and understanding of mental health. This emphasizes the role of the host country in the promotion of the mental health of refugee children. Interventions aimed to improve peer relations and to reduce discrimination are needed, and these point to the school as a potential arena for positive change. Parental support groups were also mentioned as helpful in understanding the children’s need for support. It should not be surprising that any attempts to improve and maintain the mental health of refugee children should be underpinned by parental support.

## Data Availability Statement

Requests to access the data should be directed to Fatumo Osman (fatumo.osman@pubcare.uu.se). 

## Ethics Statement

Ethical approval for the study was obtained by the Uppsala Regional Ethics Committee (Etikprövningsnämnden) in December 2018 (2018-12-05: Dnr 2018/486). Invited participants received information about the purpose of the study and the research method verbally and through information sheets prior to interview. The contact details of the researcher were given to the participants for any further questions prior or after the interview. The participants were also informed about their right to withdraw their consent without penalty and that confidentiality would be maintained. Written informed consent was obtained from participants for their participation and to publish the study in a scientific paper.

## Author Contributions

AA made all the contacts with the participants, performed the interviews, and led the analytic process. AA and FO analyzed the results, and AS and GW contributed to the study design and verified the findings of the analysis. All authors contributed to writing the paper.

## Funding

This study was funded by the Kavli Trust (Grant ID: A-321629). The funder has had no involvement in the design of the study, data collection, analysis or interpretation, or the writing of the manuscript.

## Conflict of Interest

The authors declare that the research was conducted in the absence of any commercial or financial relationships that could be construed as a potential conflict of interest.
